# Altered RV Mechanics Post-LVAD Insertion: a Physiological Perspective!

**DOI:** 10.21470/1678-9741-2020-0058

**Published:** 2020

**Authors:** Rohan Magoon, Jes Jose, Jasvinder Kaur Kohli, Ramesh Kashav

**Affiliations:** 1 Department of Cardiac Anaesthesia, Atal Bihari Vajpayee Institute of Medical Sciences (ABVIMS) and Dr. Ram Manohar Lohia Hospital, Baba Kharak Singh Marg, New Delhi, India. E-mail: rohanmagoon21@gmail.com; 2 Department of Anaesthesia, Atal Bihari Vajpayee Institute of Medical Sciences (ABVIMS) and Dr. Ram Manohar Lohia Hospital, Baba Kharak Singh Marg, New Delhi, India.

Intensive care of patients on left ventricular assist device (LVAD) support is compounded by the peculiar physiological consequences of circulatory assistance.

**Fig. 1:**
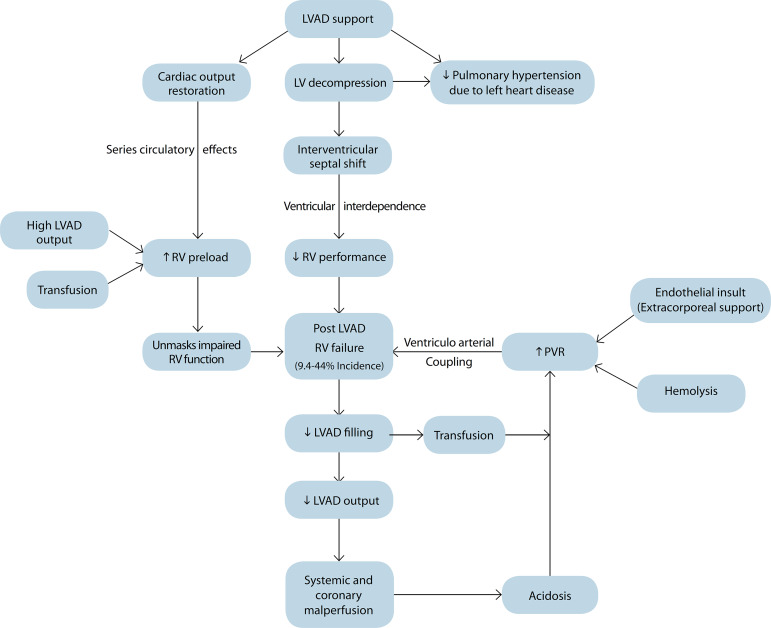
The pathophysiology of post-left ventricular assist device (LVAD) right ventricular (RV) dysfunction. LV=left ventricular; PVR=pulmonary vascular resistance

In this context, clinical deterioration in the right ventricular (RV) function is often encountered post-LVAD implantation, with an incidence of 9.4-44% and is associated with an elevated risk of mortality^[1,2]^. The key principles determining the RV mechanical alterations are *series circulatory effects, ventricular interdependence, and ventriculoarterial coupling* (Figure 1). Firstly, LVAD assistance rapidly restores the cardiac output, resulting in an augmented RV preload owing to a series circulation. Secondly, a rapid offloading of the left ventricle induced by the assist device results in a leftward interventricular septum shift, significantly reducing the septal contribution to RV contractility. This geometrical alteration and the consequential architectural disadvantage by the virtue of ventricular interdependence in background of an enhanced preload can unmask pre-existing RV dysfunction^[3]^. Therefore, subtle pre-LVAD RV dysfunction is an important determinant of subsequent significant contractile impairment culminating as RV failure.

Interestingly, the principle of ventriculoarterial coupling is central to the post-LVAD RV dynamics, with any degree of elevation in pulmonary vascular resistance proving to be detrimental in such scenario. This explains the rationale behind the administration of phosphodiesterase inhibitors and other non-pharmacological and ventilatory manipulations aimed at attenuating an elevated RV afterload.

The mortality attributable to post-LVAD RV failure is largely a consequence of decreased flow to the LVAD, leading to a significant reduction in pump output, compromising the endorgan perfusion^[4]^. The coexistent systemic venous congestion compounds the situation furthermore. In the most extreme form, the clinical situation mandates biventricular assistance. Therefore, the echocardiographic predictors of post-LVAD RV failure continue to be actively investigated in order to adequately and consistently define the involved risk. However, the role of serial echocardiography in assessing the degree of septal shift on LVAD support, tricuspid valvular competency, pulmonary arterial pressure, and the concomitant RV function with the titration of inotropic support cannot be overemphasized.

The aforementioned elucidation of the impact of altered RV mechanics on clinical outcomes post-LVAD insertion highlights the importance of a sound understanding of the underlying pathomechanisms as the basis of echocardiographic surveillance and subsequent therapeutic measures. This discussion is particularly pertinent in the current era of mechanical circulatory assistance, wherein LVAD support continues to evolve as a bridge to transplantation, destination therapy, or as a temporary circulatory support with an expectant recovery of the cardiac function for the ever-growing cohort of patients with advanced heart failure, exceeding the availability of potential organ donors by a considerably wide margin^[5]^.
